# Characterization of 3D Printed Polylactic Acid by Fused Granular Fabrication through Printing Accuracy, Porosity, Thermal and Mechanical Analyses

**DOI:** 10.3390/polym14173530

**Published:** 2022-08-28

**Authors:** Luca Fontana, Alberto Giubilini, Rossella Arrigo, Giulio Malucelli, Paolo Minetola

**Affiliations:** 1 Department of Management and Production Engineering (DIGEP), Politecnico di Torino, Corso Duca degli Abruzzi 24, 10129 Torino, Italy; 2 Integrated Additive Manufacturing Centre (IAM@PoliTO), Politecnico di Torino, Corso Duca degli Abruzzi 24, 10129 Torino, Italy; 3 Department of Applied Science and Technology (DISAT), Politecnico di Torino, Corso Duca degli Abruzzi 24, 10129 Torino, Italy

**Keywords:** 3D printing, Fused Granular Fabrication, PLA, pellets, sustainable manufacturing

## Abstract

Fused Granular Fabrication (FGF) or screw-extrusion based 3D printing for polymers is a less diffused alternative to filament-based Additive Manufacturing (AM). Its greatest advantage lies in superior sustainability; in fact, polymer granules can be used to directly feed an FGF printer, reducing the time, cost and energy of producing a part. Moreover, with this technology, a circular economy approach involving the use of pellets made from plastic waste can be easily implemented. Polylactic Acid (PLA) pellets were processed at different printing speeds and with different infill percentages on a customized version of a commercial Prusa i3 Plus 3D printer modified with a Mahor screw extruder. For the characterization of the 3D printed samples, rheological, thermal, mechanical and porosity analyses were carried out. In addition, the energy consumption of the 3D printer was monitored during the production of the specimens. The results showed that a higher printing speed leads to lower energy consumption, without compromising material strength, whereas a slower printing speed is preferable to increase material stiffness.

## 1. Introduction

After 2009, when Stratasys’ major patent for Fused Deposition Modelling (FDM) process expired, a slew of cheap 3D printers for Fused Filament Fabrication (FFF) emerged on the market. Both for FDM and FFF approaches, commonly referred to as 3D printing, the material is loaded onto the machine in the form of thermoplastic filaments that are extruded through a nozzle and deposited layer-by-layer on a printing platform [1]. Despite the ever-growing popularity of 3D printing due to relatively inexpensive material and equipment costs, the requirement of an intermediate polymer filament production step causes significant inefficiencies in the production process of 3D printed objects. Therefore, a more cost- and money-efficient approach involves the use of pellets directly as raw material for 3D printing, rather than for filament production. This alternative solution is presented in the literature to solve the aforementioned drawbacks, and the pellet-extrusion technology for layered manufacturing is known as Fused Pellet Manufacturing or Modelling (FPM), Fused Granular Fabrication (FGF) or Pellet Additive Manufacturing (PAM). The main benefits of FGF compared to FFF are based on avoiding the filament manufacturing and therefore the possibility of altering polymer properties during the fabrication due to heating, but also avoiding the tight dimensional controls of the filament diameter request to minimize warping, creep or blocking of material in the 3D printer’s feed mechanism [2]. Moreover, the storage of pellets is easier than for filaments, which are wound into spools, causing a waste of material. In particular, the initial section of the filament, which is wound with a narrower winding radius, is subjected to residual stress that can embrittle less ductile materials. Two other limitations of filament-based 3D printing are the limited choice of thermoplastic polymers available on the market, as well as their price, which is at least five times higher than polymer pellets [3]. Moreover, by eliminating the filament creation process, it is much easier, after a simple pelletizing step, to directly recycle plastic waste on an AM process, following a circular economy perspective [4].

Nowadays, the Arburg Freeformer is one of the few pellet-feeding devices among industrial AM systems. A plasticizing screw melts the material in the Arburg Plastic Freeforming (APF) process, whose deposition in droplet form is metered using a piezoelectric nozzle [5]. In previous scientific research, two forms of FGF have been hypothesized and examined: a plunger-based and a screw-based one. The former uses a device similar to a syringe; for instance, Volpato et al. extruded molten polypropylene (PP) grains from a heated reservoir employing a cylinder-piston system [6]. However, the majority of researchers employed the screw-based approach for constant feeding of the 3D printer.

Reddy et al. investigated the key impact of FGF process parameters on different final properties of 3D printed objects, such as their mechanical strength and the quality of the surface [7]. An experimental screw-type extrusion system was realized by Valkenaers et al., and they applied it with a 0.2 mm nozzle for printing polycaprolactone (PCL) [8]. Tseng et al. designed and constructed a similar system that could reach very high temperatures for processing, even technopolymers, e.g., polyether ether ketone (PEEK) [9]; whereas Whyman et al. presented a machine that was also equipped with an automatic feeder [10]. Woern et al. looked at the possibility of using recycled particles for four different thermoplastics [11]. Reich et al. did a similar study on polycarbonate (PC) waste [12]. Liu et al. designed a two-stage extrusion technique to boost machine capacity and expand the use of FGF technology to the fabrication of large components. In the first step, a typical polymer extrusion plasticizing unit is employed to feed the following dosing and printing stage [13]. Nieto et al. used a screw extruder to print big acrylonitrile butadiene styrene (ABS) pieces for the naval industry’s toilets [14]. Wang et al. created an industrial extrusion system solution that can be integrated with an industry robot using multiphysics modeling software. [15]. Byard et al. used Fab Labs as 3D printing waste recycling centers to prove the environmental and financial viability of employing FGF for large-scale products [16]. Similarly, Shaik et al. demonstrated the economic advantages of using pellets as raw material for the polymer additive manufacturing process [3], whereas Alexandre et al. demonstrated that FGF has comparable mechanical performance to FFF samples [4]. Little et al. showed that rPET flakes have potential as DRAM (Distributed Recycling Additive Manufacturing) feedstock for FFF and FGF processes [17].

It is noteworthy that FGF process, as well as FDM, could be affected by some defects such as void formation [18], especially leading to a decrease in material density and mechanical performance. In their study, Ferretti et al. pointed out that the formation of pores can be a consequence of the printing strategy employed [19], and X-ray computed tomography inspection (X-CT) or in situ monitoring system during printing can be used to discover this type of defect. X-CT is a non-destructive method to visualize the interior pattern of solid objects and gain digital information about their 3D geometries and properties, by visualizing for example the presence of internal voids, such as air bubbles, in samples generated during the 3D printing process [20,21]. Minetola et al. used a low-cost camera system and a computer vision algorithm to compare the printed layer to the nominal one for detecting defects during production [22].

Computed tomography has been used by Zhang et al. for confirming the internal structure, such as the shape and dimensions of pores and of D-TMPS (Diamond-Triply Periodic Minimal Surfaces) produced by FDM technology [23]. X-CT was used by Zekavat et al. to investigate the effect of fabrication temperature on FDM printing accuracy [24]. Zhang et al. used this technique instead to inspect the inside structures of samples produced, evaluating the influence of three different materials and four infill strategies [25]. The impact of FDM process factors on the mechanical and fracture characteristics of 3D printed samples were investigated by Webbe Kerekes et al. using CT scanning to observe the failure progressions of the samples [26].

Here, a customized version of a low-cost 3D printer was employed to investigate the processability of PLA through a screw-based extrusion approach. Different rheological, thermal and mechanical characterizations were conducted to assess the best printing parameters of the material. Then, some more complex structures, e.g., chess pieces, were produced and CT-scanned to explore the inside structures and to perform a porosity analysis of the fabricated samples. To assess the printing process accuracy, a geometrical differences analysis was carried out considering as reference samples the CAD models of the 3D printed objects. Finally, to obtain a complete balance of the production process sustainability, the energy consumption required to produce different geometries was also evaluated by varying the printing speed and infill percentage. These results were then related to the previously evaluated mechanical properties. This research work aims to combine the advantages of using an eco-friendly material such as the biopolymer PLA, along with a manufacturing process that can be sustainable in terms of time, energy and price.

## 2. Materials and Methods

**Figure 1:**
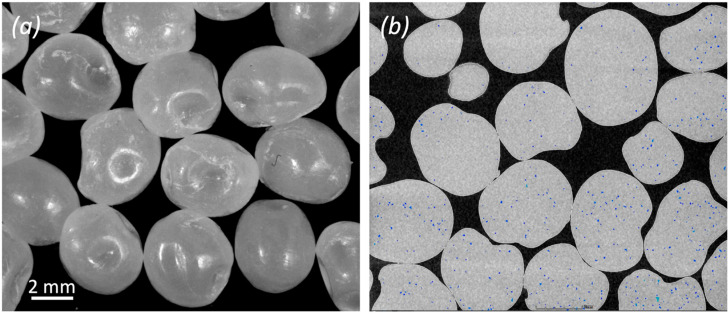
Morphology of the PLA feedstock: (**a**) Optical microscopy image of PLA pellets; (**b**) X-CT two-dimensional image of the internal structure of PLA pellets. The blue points represent internal microporosities of volume lower than 0.001 mm^3^.

Mahor XYZ (Mahor XYZ - IAMTECH 2019, Andosilla, Navarra, Spain) provided the natural PLA pellet feedstock, as well as the pellet extruder. The granules have a main dimension of about 4 mm and an irregular bean shape with the presence of small craters, as shown in Figure 1a. Their internal porosities are shown in the X-ray image of Figure 1b. The PLA pellets cost 8.8 €/kg, which is less than half the cost of a 1 kg spool of colored PLA. PLA density is 1.24 g/cm^3^ from the material datasheet.

### 2.1. Viscosity Characterization

The rheological behavior of neat PLA pellets was assessed to provide a viscosity reference value in order to determine the screw extruder’s capacity. An ARES (TA Instrument, New Castle, DE, USA) strain-controlled rheometer in parallel plate geometry was used with a 25 mm plate diameter configuration. The analyses were performed under a nitrogen environment, with frequency varying from 10^−1^ to 10^2^ rad/s at various temperatures. Each sample’s strain amplitude was chosen to fall inside the linear viscoelastic area.

### 2.2. DSC Analysis

A QA1000 TA Instrument equipment (New Castle, DE, USA) was used to conduct DSC studies. All the tests were carried out on 8 mg samples sealed in aluminium pans, and with a dry N_2_ flow of 20 mL/min. A heating ramp from 0 °C to 200 °C at 10 °C/min was used to test the PLA material.

Differential Scanning Calorimetry (DSC) was performed to calculate the degree of crystallinity (X_c_) and the thermal transitions, i.e., the glass transition temperature (T_g_), crystallization temperature (T_c_) and melting temperature (T_m_). Equation (1) was used to find the percentage of the crystalline phase (X_c_).

(1) $$X_{c}=\frac{\Delta H_{m}-\Delta H_{cc}}{\Delta H_{m}^{0}}\times 100$$

where$\;\Delta H_{m}$ is the enthalpy of fusion, $\Delta H_{cc}$ is the enthalpy of cold crystallization and $\Delta H_{m}^{0}$ is the enthalpy of fusion of a purely crystalline PLA structure, which was assumed to be equal to 93 J/g [27].

### 2.3. Tensile Tests

To analyze the mechanical behavior of the PLA processed by FGF, tensile tests were performed with an Instron^®^ 5966 dynamometer (Instrom, Norwood, MA, USA) on 3D printed ASTM D638 type IV dumbbell specimens. The tensile tests were conducted according to the same standard, and specifically at a crosshead speed of 1 mm/min until deformation of 0.2%, then 10 mm/min until fracture of the sample.

### 2.4. Screw-Based 3D Printer

A Prusa i3 Plus 3D printer, modified by Fab Lab (Spazio Geco, Pavia, Italy) and equipped with a Mahor XYZ screw-based extruder for FGF technology, was used in this study. Furthermore, a 400 W power supply was installed to boost the machine’s heating capability. Eventually, the customized 3D printer can reach a nozzle temperature of 290 °C and a platform temperature of 90 °C, so that the range of polymers that can be processed in pellet form can be expanded.

**Figure 2:**
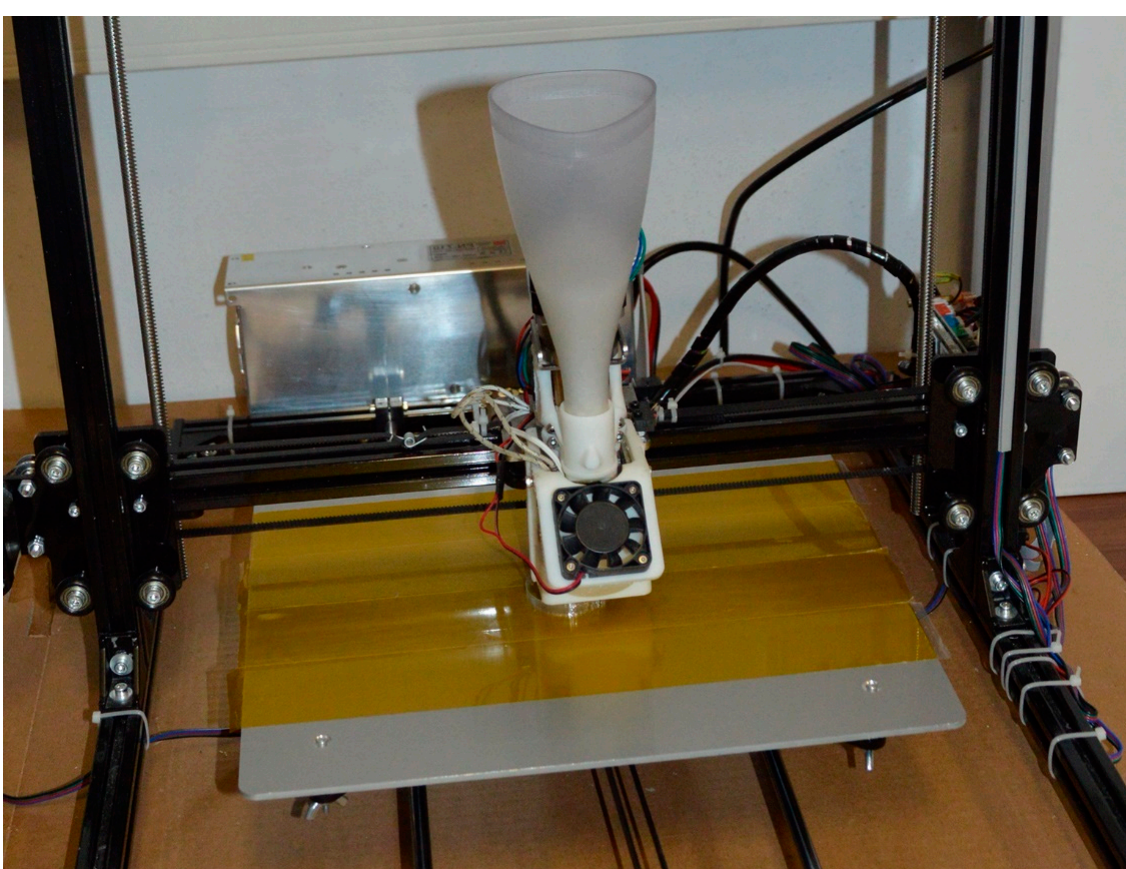
Photo of the customized FGF 3D printer with Mahor extruder.

The extruder’s hopper is a self-replicated element, like those used in other customized 3D printers [28], and the nozzle has a diameter of 0.8 mm. The original machine’s cartesian structure and axis resolution remain unaltered. Over a working volume of 300 × 300 × 420 mm^3^, the FGF printer (Figure 2) has a positioning precision of 0.004 mm for the Z axis and 0.012 mm for the X and Y axes.

### 2.5. Slicing Software

The open software Slic3r (Version 1.3.0, free open-source software developed by Alessandro Ranellucci) was chosen to slice the STL (Solid To Layer) model of the object and create the gcode printing file. The machine configuration selected in the software was replicated from the standard Prusa i3.

### 2.6. Sample Fabrication

For all printing jobs, the chosen parameters are enlisted in Table 1 and a Kapton ribbon tape was used on the platform to improve the adherence of the initial printing layer (Figure 2).

**Figure 3:**
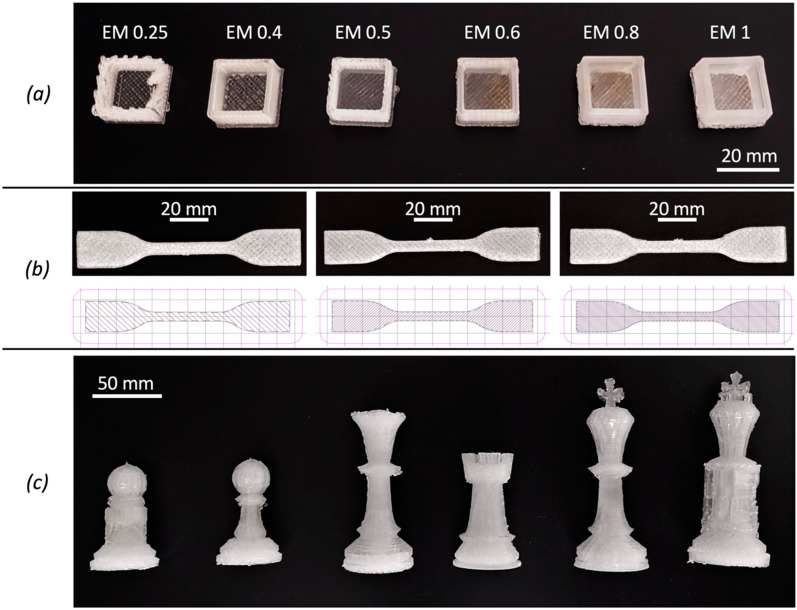
3D printed samples by Fused Granular Fabrication: test cubes printed for different values of the extrusion multiplier (**a**); dumbbell specimens and print path for 25%, 50%, 75% infill (**b**) and all produced chess pieces (**c**).

The experimental FGF 3D printer was used to fabricate different samples (Figure 3):

- 6 hollow test cubes (20 mm edge and 2 mm thickness) were realized with six different extrusion multiplier (EM) values: 0.25, 0.4, 0.5, 0.6, 0.8 and 1. EM is a dimensionless factor that modifies, in direct proportion to its value, the extrusion flow rate and thus the amount of polymer deposited by controlling the rotational speed of the screw. EM is used for fine-tuning of the deposited material for good surface finish and correct single wall width. The variation of EM was used for process optimization to determine the best printing accuracy. For all samples, 50% infill, 2 bottom and top solid layers and a high-speed printing set (Table 2) were used.
- 18 PLA tensile samples were produced varying the infill percentage as 25%, 50% and 75%. Two different printing speed sets, whose parameters are listed in Table 2, were used. For each speed set and infill value, three tensile samples were printed.
- 6 chess pieces (1 pawn, 1 king, 1 queen, 1 rook, 1 pawn with supports and 1 king with supports) were fabricated to also investigate the 3D printability of more complex shaped structures, primarily considering whether the print was successful and evaluating the deviation of each piece from its CAD model. The selected chess pieces have self-supporting geometries but also overhangs. Therefore, they were used for evaluating the printability with and without supports using a unique extruder and material. A single extruder is common for FGF machines and most low-cost 3D printers. Two different values of the infill parameter were used for the production of the chess pieces to consider the influence of the infill on the fabrication process and the final pieces as well. Specifically, an infill of 50% was adopted for the queen and the king without supports, whereas 25% infill was used for all the others.

### 2.7. X-ray Computed Tomography (X-CT)

A micro-CT scan model Phoenix v|tome|x S240 (GE Baker Hughes–Waygate Technologies, Wunstorf, Germany) was employed to inspect the 3D printed samples. The X-ray scan parameters are enlisted in Table 3, and 1500 images were acquired for each scan. For long samples, such as the tensile dumbbell or some chess pieces, more scans were needed to finish the inspection, and therefore the multi-scan function of datos|acquisition software was selected. The reconstruction of the X-ray images into a 3D model was performed with datos|reconstruction software. VG Studio Max software (version 3.4) by Volume Graphics (Hexagon Metrology–Volume Graphics, Heidelberg, Germany) was used for visualization and analysis. Before starting the porosity analysis, a surface determination was conducted with the *Advanced (classic)* approach, starting the contour from the histogram with an *Automatic* material definition at an isovalue of 50%. Based on surface determination results, the porosity/inclusion analysis module (VGDefX/Only threshold algorithm) of VG Studio software was selected for the identification of voids. For a better graphical representation of the results, in the following diagrams, the porosity volume lower than 0.005 mm^3^ and higher than the 99th percentile was excluded.

CT-scan data were also used for evaluating the dimensional accuracy of the printed parts using GOM Inspect software (version 2021).

## 3. Results

### 3.1. Viscoelastic and Thermal Characterization

**Figure 4:**
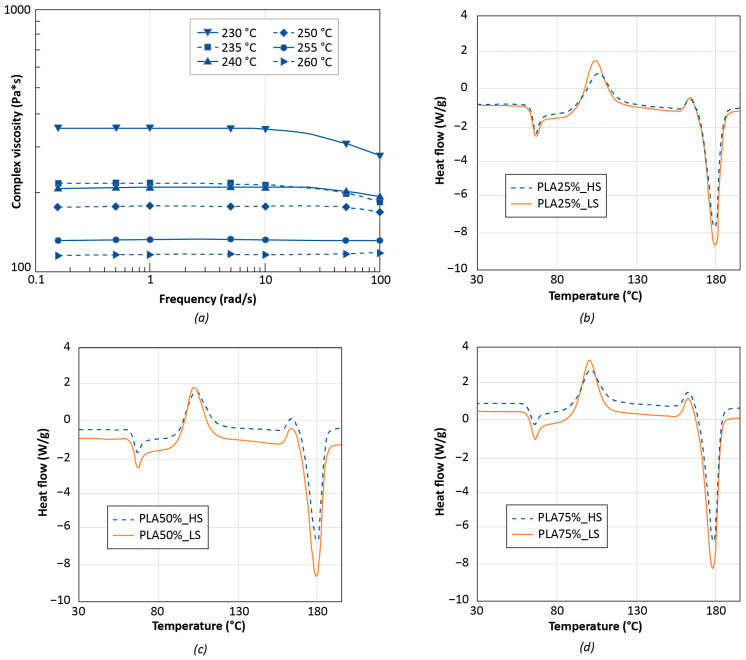
Viscosity (**a**) and DSC (**b**–**d**) curves for PLA material at different experimental temperatures.

To characterize the flow behavior of the biopolymer during the 3D printing process, rheological measurements were carried out. Analyses were conducted at different temperatures close to that of the FGF extruder to preemptively assess the flowability of the PLA material through the nozzle. The complex viscosity is plotted as a function of frequency and at various temperatures in Figure 4a.

For low values of frequency, independently from temperature, PLA first displays a Newtonian plateau followed by shear-thinning behavior that is marked by a fall in viscosity for a frequency greater than 10 rad/s.

The Newtonian behavior becomes increasingly evident as the temperature rises, and at 260 °C, it spans the whole frequency range studied. This behavior can be due to the increased mobility of polymer macromolecules at high temperatures, which thus involves the anticipation of the macromolecular chain relaxation [29]. Overall, the PLA material supplied by Mahor can be considered 3D printable because its viscosity values are within an acceptable range for an extrusion-based 3D-printing approach [30,31].

Thermal characterization of the polymer was conducted before and after 3D printing to evaluate how this manufacturing process and its parameters, such as the printing speed, may affect the structure and properties of the PLA material. Figure 4b–d and Table 4 summarize the most important thermal parameters gathered during the second heating scan. As a result of the 3D printing method, all samples have lower T_cc_ values than unprocessed PLA, implying that PLA macromolecules have a higher crystallization potential.

In terms of crystalline phase content, unprocessed PLA is mostly amorphous, but after processing PLA, its degree of crystallinity increases, owing to the orientation of polymer chains during processing, which favors the creation of crystalline structures [32].

### 3.2. Selection of Optimal Extrusion Multiplier Parameter

**Figure 5:**
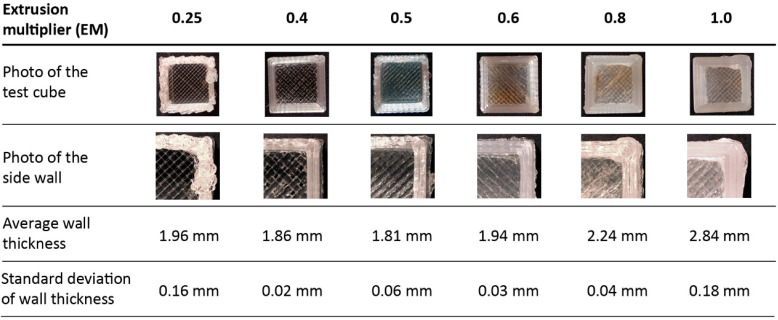
Photos and dimensional deviations of the wall thickness for the hollow test cubes 3D printed with different EM values.

The hollow test cubes were used for selecting the optimal value of the extrusion multiplier in Slic3r software. GOM Inspect software (GOM GmbH, Braunschweig, Germany) was used to obtain an accurate measurement of the wall thickness of the printed cube samples, which was compared to the nominal thickness of the CAD model. To this aim, the CT scan data of each cube was imported in STL format in GOM Inspect and the built-in function of Construct Outer Disc Caliper was used to measure the thickness of the side wall of each cube in more than one position. This function works as a virtual caliper for measuring the distance between two parallel discs in contact with the element being measured. The software creates two touch points at the positions where the touch discs first touch the element and then computes the distance between both touch points. The wall thickness was taken as a significant reference parameter to calibrate the EM. From Figure 5, it can be observed that the hollow test cube with EM 0.6 has the minimum deviation from the nominal value of the wall thickness, without considering the hollow test cube with EM 0.25. This minimum EM value is a case of under-extrusion, which does not deposit enough material, as evidenced by the low sample weight and the maximum deviation from the nominal volume of the cube (Table 5). Because of the under-extrusion for EM 0.25, the poor quality of the corresponding cube sample can also be qualitatively assessed from the magnification of the side wall in Figure 5.

It can be concluded that low values of the EM parameter reduce the flow rate, resulting in an inadequate quantity of extruded and deposited material, i.e., under-extrusion. In contrast, when the extrusion multiplier is greater than 0.6, an excess of material is over-extruded. Thus, the side walls of the test cube become excessively thick (Figure 5).

For the weight calculation of each sample, a Gibertini 1000HR-CM balance, with an accuracy of 0.01 g, was used. The results of the measurements are reported in Table 5 along with the experimental volume for each sample. The volume was calculated according to the CT analysis and compared to the nominal CAD volume of the hollow cube, which is 1.646 cm^3^. The accuracy of CT volume measurements depends on the voxel size, which in the case of the cubes is about 34 μm; therefore 4 × 10^−5^ mm^3^ is the size of the smallest volume element.

The best result in terms of minimum deviation from the nominal volume was obtained for an EM value of 0.6.

According to the previous results, this value of the extrusion multiplier was assumed as the optimal one and all 3D printed samples for the next phase of this research were produced with an EM of 0.6 for the slicing operation in Slic3r software.

The characterization of the 3D printed hollow test cubes is finalized with a porosity analysis using their CT scan data. The AM process is affected by the presence of voids and is possibly due to the presence of moisture in the polymer pellets, which evaporates during the printing process, leading to the formation of micropores [20]. However, in the case of the FGF process, part porosity might also be a consequence of a discontinuous material extrusion due to the inherent geometry of the pellet feedstock, which is not continuous as the filament of traditional 3D printing.

**Figure 6:**
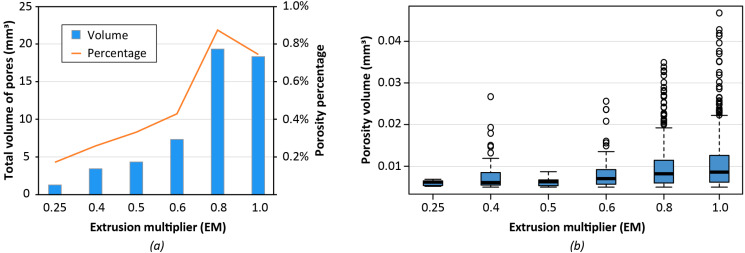
Porosity analysis of hollow test cubes 3D printed with different Extrusion Multiplier (EM) values: (**a**) total volume of pores for different values of EM; (**b**) distribution of porosity volume at varying EM (excluding pores with volume lower than 0.005 mm^3^ and higher than the 99th percentile).

The results of the porosity analyses are presented in Figure 6, where the total volume of porosity is expressed as a function of EM, along with its distribution for all six different values of EM. From the analysis of the plots, the highest values are found for EM equal to 0.8 and 1, with a significant distinction from the cases with EM less than or equal to 0.6 (Figure 6a). This result can be attributed to the higher amount of deposited material that increases by increasing EM. Thus, the total volume of pores rises. Moreover, from the distribution of the pore volume in Figure 6b, it can be observed that the sample with EM 0.6 has a lower median pore volume than the samples with EM 0.8 and EM 1.

The porosity percentage, calculated as a ratio between the total volume of the object and the total pores volume, is slightly higher for the EM 0.6 sample if compared to one of the samples with lower EM. However, the dimensional accuracy of samples with lower EM is worse. Therefore, the value of 0.6 for the extrusion multiplier represents the best trade-off considering both porosity and dimensional analyses that were previously presented.

### 3.3. Mechanical Characterization

Dimensional characterization after 3D printing was also conducted for the tensile samples. The dumbbell samples were weighted with the Gibertini balance and their average thickness was measured with a micrometre.

The results are enlisted in Table 6 with the printing time and the energy consumption of specimen production, which was measured using a Meterk M34EU power meter plug.

**Figure 7:**
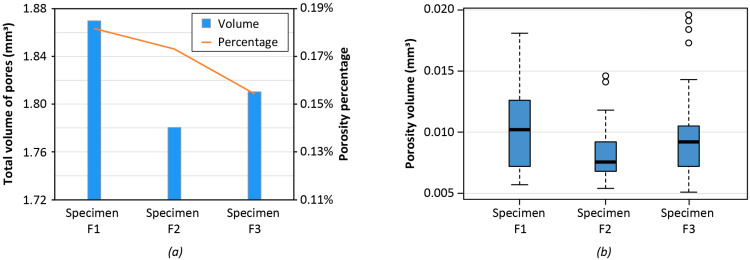
Porosity analysis of 3 dumbbell specimens: (**a**) total volume of pores for 3 different tensile samples produced with LS printing set and 25% infill; (**b**) distribution of porosity volume at varying EM (excluding pores with volume lower than 0.005 mm^3^ and higher than the 99th percentile).

The three dumbbell samples with 25% infill for the LS printing set were CT-scanned before tensile testing. The CT analyses were aimed at evaluating the repeatability of the AM process for the tensile samples with specific reference to the presence of porosities. Figure 7 illustrates the porosity results that are consistent for all three specimens with the porosity percentage ranging from 0.14% to 0.18% (Figure 7a). The pore distribution (Figure 7b) also confirms that the statistics are similar for the considered samples.

**Figure 8:**
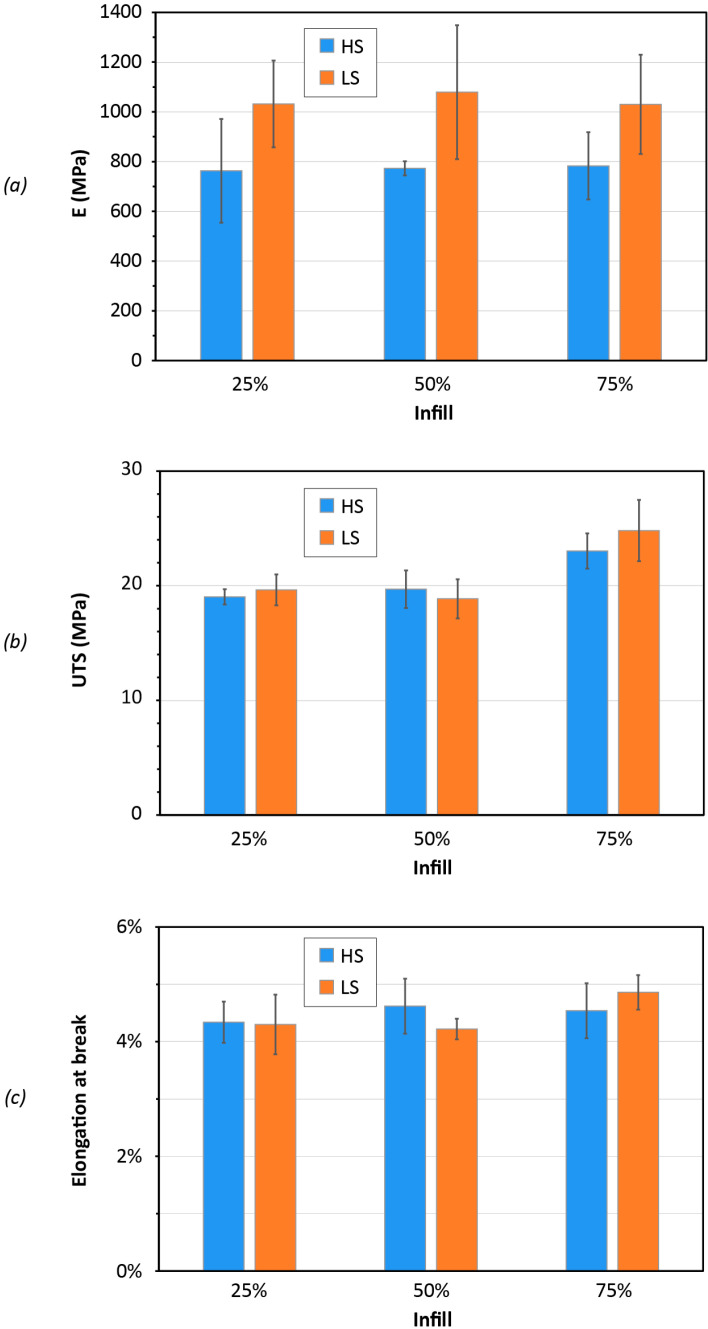
Mechanical performance of the dumbbell specimens for three different infill percentages (25%, 50%, 75%) and the two printing-speed sets of high speed (HS) and low speed (LS): elastic modulus (**a**); ultimate tensile strength (**b**) and elongation at break (**c**).

The results of the tensile tests for the 3D printed specimens are summarized in Figure 8. The main mechanical characteristics of PLA from FGF are expressed in terms of Young’s modulus (E), ultimate tensile strength (UTS) and elongation at break. It can be noted that the processing parameters have little effect on the UTS and the elongation at break. PLA specimens produced with low printing speed (LS) revealed a stiffer behavior, which is represented by a higher value of the elastic modulus.

The results of tensile tests for the FGF samples are in line with those that were previously presented in the literature for PLA material. 

A recent work by Wang et al. [33] can be taken as a reference for a comparison with the traditional 3D printing or FDM process. The FGF-printed PLA material demonstrated a slightly lower tensile strength (UTS) than the 3D printed PLA from filament feedstock. However, the reasons for this difference cannot be easily determined, as the two processes are different and do not use the same material grade and printing parameters.

For this study, the tensile test results can also be associated with the DSC outcomes in Table 4. Except for the specimens with 50% infill, the samples printed with the LS parameter set had a slightly lower degree of crystallinity X_c_ than those fabricated with the HS settings. It is well known that in crystalline regions, the polymer chains have a compact structure which endures the stress and material resistance. Conversely, in the amorphous regions, the polymer chains are loose and can be easily deformed providing more contribution to the strain. This aspect can be distinguished in the results of the ultimate tensile strength (Figure 8b), wherein the specimens with 75% infill, that have a higher X_c_ value, demonstrated greater resistance to break. Specimens with 75% infill had a UTS of about 25 MPa, whereas all other specimens had an ultimate resistance slightly lower than 20 MPa. This result can be ascribed to a combination of both the higher degree of crystallinity and thicker printing layer for the specimens with 75% infill.

In the case of the elongation at break (Figure 8c), for the same infill percentage, the specimens with a higher X_c_ value demonstrated a slightly smaller strain. However, it should also be remarked that the difference in the crystallinity percentage is quite small for the same infill percentage and a different printing speed setting.

The increase in the material stiffness was not observed when the degree of crystallinity was higher. Independently from the infill percentage, all tensile specimens printed with the LS parameters had an elastic modulus E of about 1000 MPa. The tensile specimens printed with the HS parameters had an elastic modulus E close to 800 MPa (Figure 8a).

Further investigation would be needed to deepen the analysis of size and structure for crystalline regions because these factors can affect the mechanical behavior of the PLA material [34].

### 3.4. Fabrication and Characterization of Chess Pieces

A reduced set of four chess pieces was selected as a case study for the production with the FGF printer of small parts with more complex geometry than the previous samples. The optimal value of EM 0.6 was used for the fabrication of the chess set as well. The set included a pawn, a rook, the queen and the king. All parts were 3D printed without the use of supports. The pawn and the king were also produced with supports for overhanging geometries, such as the cross on the top of the king. The replicas with supports are used to assess the influence of the support structure on the dimensional and geometric accuracy of FGF products. However, supports are printed with the same material as the parts, so additional post-processing and manual operations are needed for their removal. From this point of view, an FGF printer is similar to a traditional 3D printer with a unique extruder and nozzle, which does not allow the user to print the support structure using another material different from the one of the product.

Table 7 summarizes the weight of the six chess pieces along with the measures of the energy consumed by the FGF printer for their production and the required printing time.

Further analyses of the 3D printed chess pieces were conducted using the CT-scan data. After CT-scanning, the CT data of the chess pieces was first imported and aligned to the corresponding original CAD model using the best-fit function in GOM Inspect environment. After the alignment, the deviations between the actual piece geometry of CT data and the nominal geometry of the CAD model were computed. A range of +/−2.5 mm was chosen for all chess pieces as a reference search distance to get comparable results for the dimensional deviation on the piece surface.

**Figure 9:**
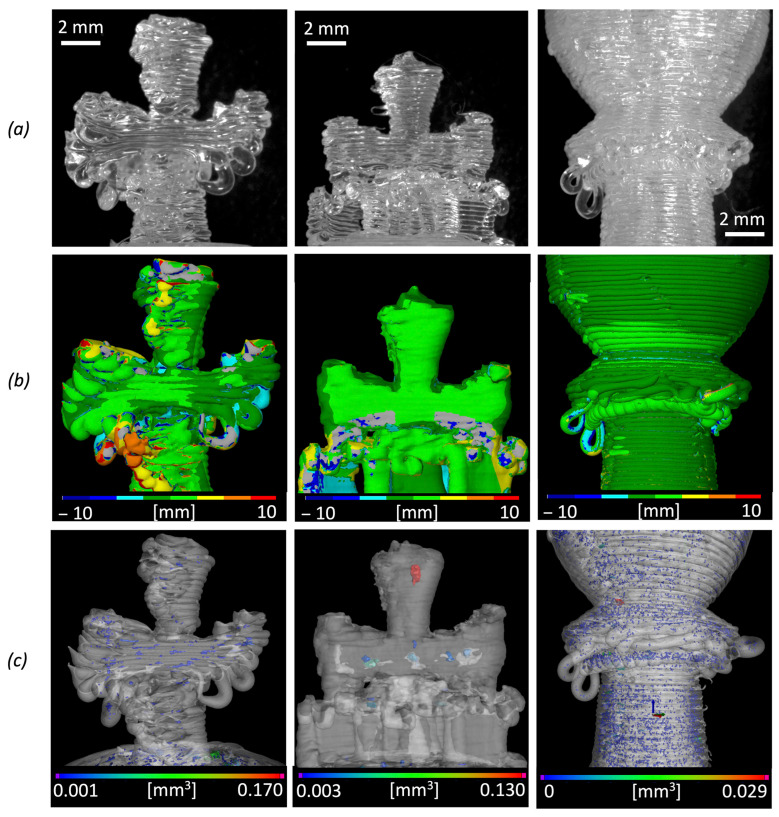
Representation of local defects for different chess pieces produced by FGF: cross detail of the king without support, cross detail of the king with supports and collar detail of the pawn without support. (**a**) Optical microscopy images; (**b**) GOM Inspect analysis results; (**c**) volume distributions of internal microporosities obtained by X-ray CT characterization.

The results of data comparisons and their deviations are reported in Table 8. For the printed pieces, the average distance from the nominal CAD surface is around −0.39 mm. Larger dimensional and geometric errors can be due to some FGF printing defects, such as filaments squeezing during material deposition of filament collapse or folding for unsupported overhangs. Figure 9 highlights some examples of these local defects of the 3D printed chess pieces. In this figure, the optical microscopy images (Figure 9a) of the real pieces are compared to the coloured deviation maps (Figure 9b) in the area of major printing defects. The effect of the presence of supports over the definition and dimensional accuracy is particularly significant for the king’s cross.

**Figure 10:**
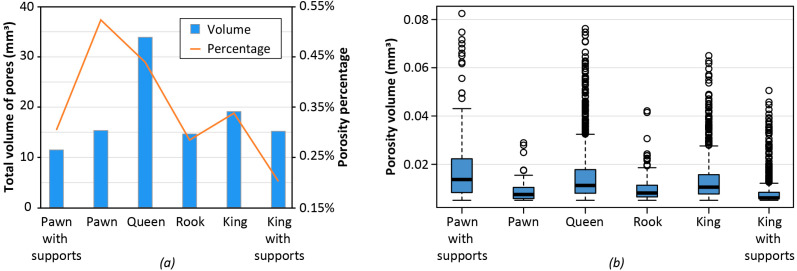
Porosity analysis of a complete replica of 3D printed chess pieces: (**a**) total volume of pores for the 3D printed chess pieces; (**b**) distribution of porosity volume at varying EM (excluding pores with volume lower than 0.005 mm^3^ and higher than the 99th percentile).

The internal defects of all chess pieces were assessed through CT scanning, and the local distribution of some internal microporosities can be observed as an example in Figure 9c. Figure 10 summarizes the results of the porosity analysis from CT data. A comparison between the results for the two pawn pieces with and without supports is particularly interesting. The pawn with supports has a lower total volume of pores (Figure 10a). However, for this piece, the median volume of the single pore is larger than the median volume of the single pore of the pawn without supports (Figure 10b). This means that the pawn without supports has a wider number of pores (porosity percentage of 0.52%) but their volume is small.

The effect that printing infill has on porosity is noteworthy. Assuming that under normal operating conditions the FGF process is affected by a certain fixed percentage of defects per unit volume of deposited material, larger and heavier parts should have a greater total volume of pores. This statement is validated by the two chess pieces of the queen and the king. These pieces were printed with 50% infill and have the largest total pore volume if compared to the other pieces that were fabricated with 25% infill.

This aspect also justifies the differences between the king without supports and the king with supports. The king without supports was printed with 50% infill, whereas 25% infill was used for the king with supports. The deposited mass for the former is 9.87 g, while a mass of 9.80 g is obtained for the latter because of the support presence. For this reason, the king without supports has a greater total volume of pores than the king with supports. However, there are also exceptions and there is not a direct relationship between the total volume of pores and the deposited mass. For example, the pawn without supports, which has the smaller mass, has the greatest porosity percentage and a total volume of pores which is comparable to the one of the rook and also larger than the one of the pawn with support. Further analyses would be needed to better identify the origin of pores and explain the differences in specific cases.

## 4. Conclusions

In this research work, different geometries were realized with PLA pellet feedstock using a screw extrusion-based FGF machine after choosing the optimal extrusion multiplier parameter to tune the material flow rate in the slicing software. Considering the extrusion temperature of the polymer pellets of about 245 °C, the material showed a viscosity of roughly 200 Pa·s, which allowed proper dosing and operation of the Mahor extruder. The rheological examination of the PLA material yielded a reference value that will be of relevant interest for future investigations in the field of 3D printing sustainability of the FGF technology with different polymer granules.

The outcomes of the mechanical characterization of FGF-printed PLA showed that the highest ultimate tensile strength was reached for the 75% infill. On the other hand, no significant variation in UTS was found between 25% infill and 50% infill of PLA samples. The study of the relationship between porosity and mechanical properties of FGF printed part was beyond the scope of this paper, but it could be tackled in future research activities.

Concerning porosities, the more material extruded, the higher the probability of the presence of micropores. This result is intrinsically related to the FGF process, as discussed earlier. Printed parts with 50% infill, such as the test cubes or the queen and king chess pieces, had a porosity percentage above 0.35%. All printed samples with an infill of 25% had a porosity percentage below 0.30%, and the minimum percentage of about 0.17% was reached by the three tensile samples.

Comparing the energy required to print the various samples with the actual mechanical performance obtained from the tensile tests could provide important insight into the eco-efficiency of the customized 3D printer based on a Fused Granular Fabrication approach. Since the value of UTS was found to be unaffected by the printing speed set, higher printing speeds are beneficial to completing the production in less time, resulting in cost and energy savings. When dividing the energy consumption to print each tensile specimen by the value of the related UTS, about 0.01 MJ/MPa is needed in high-speed printing conditions (HS), whereas for the low-speed configuration (LS), the energy consumption is almost double. Printing the sample with 25% infill yields the maximum efficiency of 0.008 MJ/MPa using the high-speed configuration.

The scenarios opened by this study are promising for the design of new research activities aimed at testing the capability of the customized FGF 3D printer with different kinds of bio-matrix thermoplastics or recycled plastic goods. In this way, the advantages of a green material for its formulation and disposal can be combined with the benefits of sustainable processing to consider all perspectives of circular economy. In addition, this new AM technology enables the possibility of generating polymer blends directly in the FGF extruder in order to customize the final performance of the part according to the needs of the end user and its field of application.

## Figures and Tables

**Table 1:** Printing parameters established on Slic3r software.

Parameter	Value
Extruder temperature	245 °C
First layer bed temperature	65 °C
Layer bed temperature	60 °C
Fill pattern	Rectilinear
Fill angle (referred to the X axis)	−45°/+45°
Layer height	0.3 mm
Number of bottom solid layers	2
Number of top solid layers	2
Number of perimeters	1

**Table 2:** Speed parameters of the two printing sets.

Parameter	High-Speed Printing Set (HS)	Low-Speed Printing Set (LS)
Speed for perimeters [mm/s]	15	7.5
Infill speed (internal and top solid one) [mm/s]	15	7.5
Infill speed for solid and gaps [mm/s]	20	10
Speed for bridges [mm/s]	30	15
Speed for support material (skirt) [mm/s]	30	15
Speed for non-print moves [mm/s]	150	100
First layer speed [mm/s]	15	7.5

**Table 3:** X-ray scan parameters.

Sample	Voxel Size [μm]	Voltage [kV]	Current [μA]	Scanning Time [min]
PLA granules	17	210	85	40
Test cubes	34	160	160	40
Tensile specimens	38	160	160	45
Pawn with supports	44	180	140	22
Pawn	31	160	160	50
Queen	40	160	160	50
Rook	36	160	160	50
King	41	160	160	50
King with supports	49	180	140	50

**Table 4:** Results of the DSC analysis.

Sample	ΔH_cc_ [J/g]	ΔH_m_ [J/g]	X_c_ [%]
Unprocessed PLA	27.1	30.8	3.98
PLA25%_LS	36.7	50.2	14.5
PLA25%_HS	31.9	47.0	16.2
PLA50%_LS	38.3	52.5	15.3
PLA50%_HS	37.4	51.0	14.6
PLA75%_LS	37.9	58.3	21.9
PLA75%_HS	34.9	57.5	24.3

**Table 5:** Characterization of weight, printing time and energy consumption for hollow cubes fabricated with different values of the Extrusion Multiplier (EM).

Sample	Mass [g]	CT Volume [cm^3^]	Experimental Density [g/cm^3^]	Deviation from Nominal Volume [cm^3^]
EM 0.25	0.95	0.721	1.321	−0.925
EM 0.4	1.70	1.315	1.292	−0.331
EM 0.5	1.68	1.296	1.295	−0.350
EM 0.6	2.19	1.692	1.295	0.047
EM 0.8	2.81	2.167	1.297	0.521
EM 1	3.16	2.437	1.298	0.791

**Table 6:** Characterization of thickness, weight, printing time and energy consumption for the fabrication of tensile specimens using the modified printer with the Mahor extruder.

Sample	Infill [%]	Printing Speed Set	Thickness [mm]	Average Thickness [mm]	Mass [g]	Average Mass [g]	Printing Time [min]	Energy Consumption [MJ]
A1	75	HS	4.10	4.10	4.96	4.92	27	0.252
A2	75	HS	4.07		4.88			
A3	75	HS	4.12		4.91			
B1	75	LS	4.11	4.11	4.90	5.03	59	0.504
B2	75	LS	4.12		5.08			
B3	75	LS	4.11		5.11			
C1	50	HS	4.14	4.04	4.11	4.15	23	0.216
C2	50	HS	4.03		4.24			
C3	50	HS	3.95		4.10			
D1	50	LS	4.19	4.06	4.07	4.06	48	0.396
D2	50	LS	4.00		3.95			
D3	50	LS	4.00		4.14			
E1	25	HS	3.98	4.00	3.30	3.35	19	0.180
E2	25	HS	4.02		3.36			
E3	25	HS	4.01		3.39			
F1	25	LS	3.93	3.92	3.32	3.36	40	0.324
F2	25	LS	3.86		3.45			
F3	25	LS	3.98		3.32			

**Table 7:** Characterization of weight, printing time and energy consumption for the 3D printed chess pieces.

Sample	Infill	Printing Speed set	Mass [g]	Printing Time [min]	Energy Consumption [MJ]
Pawn with supports	25%	LS	5.02	59	0.427
Pawn	25%	LS	3.94	46	0.396
Queen	50%	HS	9.78	54	0.489
Rook	25%	HS	5.67	31	0.283
King	50%	HS	9.87	55	0.494
King with supports	25%	LS	9.80	115	0.833

**Table 8:** Alignment results of 3D printed chess pieces.

Sample	Max Distance [mm]	Min Distance [mm]	Average Distance [mm]	Standard Deviation [mm]
Pawn with supports	2.5	−2.5	−0.21	1.00
Pawn	2.5	−2.5	−0.39	0.73
Queen	2.5	−2.5	−0.4	0.55
Rook	2.5	−2.5	−0.38	0.7
King	2.5	−2.5	−0.77	0.9
King with supports	2.5	−2.5	−0.21	1.07
